# Contextualizing the Chronic Care Model among Non-Hispanic Black and Hispanic Men with Chronic Conditions

**DOI:** 10.3390/ijerph19063655

**Published:** 2022-03-19

**Authors:** Matthew Lee Smith, Caroline D. Bergeron, Ledric D. Sherman, Kirby Goidel, Ashley L. Merianos

**Affiliations:** 1 Center for Population Health and Aging, Texas A&M University, College Station, TX 77843, USA; caroline@bergeron.ca (C.D.B.); ashley.merianos@uc.edu (A.L.M.); 2 Department of Environmental and Occupational Health, School of Public Health, Texas A&M University, College Station, TX 77843, USA; 3 LIFE Research Institute, University of Ottawa, Ottawa, ON K1N 6N5, Canada; 4 Department of Health and Kinesiology, College of Education, Texas A&M University, College Station, TX 77845, USA; lsherman@tamu.edu; 5 Public Policy Research Institute, Texas A&M University, College Station, TX 77843, USA; kgoidel@tamu.edu; 6 Department of Communication, College of Liberal Arts, Texas A&M University, College Station, TX 77843, USA; 7 School of Human Services, University of Cincinnati, Cincinnati, OH 45221, USA

**Keywords:** minority health, chronic disease, men’s health, healthcare utilization, aging

## Abstract

Middle-aged and older men of color with chronic conditions have low utilization of preventive health services. In the context of the Chronic Care Model (CCM), the objective of this study was to identify perceptions about being informed, activated patients and having productive interactions in healthcare settings among non-Hispanic Black and Hispanic middle-aged and older men with chronic health conditions in the United States. Using an internet-based survey deployed nationally using a Qualtrics panel, data were collected from a sample of non-Hispanic Black and Hispanic men aged 40 years and older with one or more self-reported chronic conditions (*n* = 2028). Chi-square tests and one-way ANOVAs were used to describe this national sample by race/ethnicity and age group (40–64 years and ≥65 years). Results suggest that most health-related factors differed more on age than by race/ethnicity. Younger age groups reported less preventive care, greater barriers to self-care, mental health issues, and risky behavior. Findings from this study provide insight into the health status and healthcare utilization of racial/ethnic men with one or more chronic conditions. Results may help inform prevention and treatment interventions for middle-aged and older men of color.

## 1. Introduction

Approximately 60% of American adults have at least one chronic disease [1], but the prevalence varies by age, gender, and race and ethnicity. The incidence of chronic conditions increases with age, primarily because long-term exposure to unhealthy lifestyles can create chronic inflammation in the body and lead to the development of one or more diseases [2,3]. Non-Hispanic Black and Hispanic men are particularly at risk for chronic conditions. For example, data from the U.S. Office of Minority Health report that Black men are 3.2 times more likely to be hospitalized for uncontrolled diabetes and 1.9 times more likely to die from diabetes compared to non-Hispanic White men [4]. In addition, Hispanic men are 1.9 times more likely to have liver cancer and 1.4 times more likely to have chronic liver disease and cirrhosis than non-Hispanic White men [5].

A greater research focus is needed on non-Hispanic Black and Hispanic men with chronic conditions. The most relevant studies on health-care utilization of Black and Hispanic men with chronic conditions (e.g., from Dunlop et al. [6], Thorpe et al. [7], and Laditka and Laditka [8]) use older data. None provides a recent and comprehensive overview of the health indicators, preventive health practices, perceptions of health, and healthcare utilization among racially/ethnically diverse men with chronic conditions.

**Figure 1:**
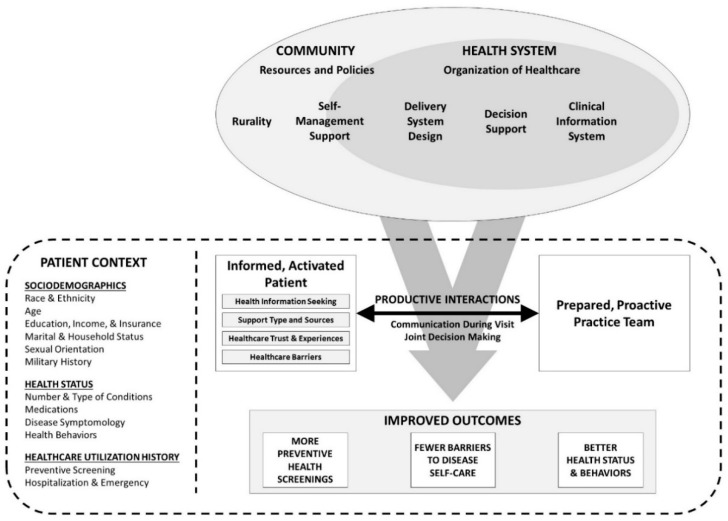
Modified Chronic Care Model Accounting for Patient Context.

Given the existing gaps in the literature related to these subgroups, the objective of this study was to identify the health status, healthcare utilization, and facilitators and barriers related to chronic disease management among middle-aged and older non-Hispanic Black and Hispanic men with chronic health conditions in the U.S. Descriptively stratifying and comparing these factors across age and racial/ethnic subgroups of men can assist in risk classification, drive future studies using advanced statistical methods, and inform risk reduction and disease prevention interventions. As seen in Figure 1, this nationwide investigation was guided by the Chronic Care Model (CCM) [9] to examine the context of chronic disease management from the patient perspective. The CCM is a widely recommended, evidence-based approach to narrow evidence-to-practice gaps regarding the prevention, diagnosis, and treatment of chronic diseases [10,11]. In systematic reviews, CCM approaches have been shown to improve intervention development, healthcare quality, and health-related outcomes [12,13]. While the original CCM was based on the six key elements [14,15] and is typically characterized by how it is implemented within healthcare settings and systems [12], CCM models have evolved to account for changes in population demographics, the U.S. healthcare infrastructure, and growing emphasis on disease prevention [16,17,18]. To further evolve the CCM, and give a unique perspective to this model for non-Hispanic Black and Hispanic men living with chronic conditions, this study utilized self-reported participant data to identify their healthcare experiences (i.e., being an informed and activated patient, interactions with healthcare providers) in the context of them as patients. Because of the nature of these data, heath system characteristics were not available. Identifying and understanding barriers to disease self-management and care among this uniquely understudied and vulnerable population are critical to help inform targeted prevention and management interventions and improve overall health outcomes.

## 2. Materials and Methods

### 2.1. Participants and Procedures

This national study utilized cross-sectional data collected from a Qualtrics Online Panels sample between September and November 2019. The internet-delivered instrument was purposively designed to capture health-related attitudes and behaviors of non-Hispanic Black and Hispanic men aged 40 years and older with one or more chronic conditions. In an attempt to comprehensively examine the factors related to health among this sample, the instrument encompassed measures represented by the CCM to include information about the participants (i.e., sociodemographics, health status, and healthcare utilization history), their interactions with healthcare professionals, and perceptions about support for and barriers to disease self-management. Additional details related to the selected measures for this study, based on the modified CCM in Figure 1, are described in the Measures section below.

Historically, racially/ethnically diverse populations are difficult to reach using traditional probability-based sampling and telephone survey research methodologies [19]. For example, telephone surveys using probability-based sampling and filtering to identify non-Hispanic Black and Hispanic men aged ≥40 with chronic conditions would be prohibitively expensive. Estimating that there are approximately 42 million non-Hispanic Black and Hispanic male adults in the United States aged over 18 years (although this study sampled men 40 years and older with one or more chronic conditions), conservative sampling estimates with a 95% confidence level and confidence interval of 3, the sample size needed for this study was 1067. 

Using Qualtrics Online Panels, researchers can effectively recruit and enroll these hard-to-reach populations to obtain nationally-representative samples [20]. After potential participants were identified by Qualtrics, they proceeded to the internet-delivered survey link, where they were presented with an Institutional Review Board-approved information sheet. Study participation was voluntary and participants could stop taking the survey at any time. Overall, 2810 potential respondents began the survey. Of these potential respondents, 2522 qualified for survey participation on the basis of three specified inclusion criteria: race and ethnicity (i.e., non-Hispanic Black or Hispanic); age (i.e., ≥40 years); and self-reporting one or more chronic conditions. Data cleaning and data quality checks were performed by Qualtrics, including removing data with incomplete or undifferentiated responses and where completion times were too quick given the scope of the data collection. Therefore, the final dataset included 2028 completed responses for an 80.4% completion rate. 

### 2.2. Measures

The survey instrument was developed by the research team and included 105 items that were identified, in part, from existing validated sources (e.g., the Behavioral Risk Factor Surveillance System [21], the National Council on Aging Chronic Care Survey [22,23], the National Study of the Chronic Disease Self-Management Program [24,25], and the Brazos Valley Health Status Assessment [26]). Generally, the instrument was divided into sections regarding participants’ sociodemographics, health indicators, healthcare utilization (preventive and treatment), behaviors, and perceptions. On average, the instrument took approximately 30 minutes for participants to complete. This study has resulted in one of the largest and most comprehensive datasets available focusing exclusively on issues related to the health of non-Hispanic Black and Hispanic men in the U.S.

#### 2.2.1. Patient Context—Sociodemographics

*Dependent Variable*. All study data were stratified and compared across the 4 category dependent variable reflecting the age groups of these non-Hispanic Black and Hispanic men (i.e., non-Hispanic Black men aged 40–64, non-Hispanic Black men aged ≥ 65, Hispanic men aged 40–64, and Hispanic men aged ≥ 65).

*Sociodemographics*. Sociodemographic measures included sex (male only), educational attainment (≤high school graduate, some college/2-year degree, ≥4-year degree), marital status (married/partnered, never married, divorced/separated, widowed), number of persons living in the household (including self), sexual orientation (straight/heterosexual, gay/homosexual, bisexual, other), annual household income level (in mostly $10,000 USD increments), health insurance coverage status (no/don’t know, yes), past/current service in the U.S. armed services (no, yes), and residential rurality status (metro, non-metro).

#### 2.2.2. Patient Context—Health Status

*Chronic Conditions*. Chronic conditions were measured using a self-reported “check all that apply” list that presented the following 19 chronic physical and mental health conditions: (1) asthma/emphysema/chronic breathing or lung problem; (2) arthritis/rheumatic disease; (3) cancer or cancer survivor; (4) chronic pain; (5) depression or anxiety; (6) diabetes; (7) heart disease; (8) high cholesterol; (9) hypertension; (10) kidney disease; (11) memory problem; (12) obesity; (13) osteoporosis; (14) obstructive sleep apnea; (15) schizophrenia or other psychotic disorder; (16) stroke; (17) thyroid problem; (18) urinary incontinence; and (19) another chronic condition not listed. Participants reported the number of different medications taken daily (range from 0 to ≥6).

*General Health Status and Disease Symptoms*. Overall general health status was assessed as a count variable with scores ranging from poor to excellent [27]. Participants also rated the following symptoms using a 0 to 10 (best possible or major problem/concern) scale separately for quality of life, fatigue, pain, shortness of breath, stress, and sleep problems [24,25]. For quality of life, 0 = worst possible and 10 = best possible. For the other measures, 0 = no problem/concern and 10 = major problem/concern.

*Depression Symptomatology*. While self-reported depression was included within the chronic conditions list, current depressive symptomatology was also measured using the Patient Health Questionnaire-2 (PHQ-2), which is comprised of two questions about feeling depressed and little interest in doing usual activities [28,29]. The PHQ-2 scores range from 0 to 6, and this study used the recommended cut-off point to distinguish between no current depressive symptomatology (0–2 score) and current depressive symptomatology (3–6 score).

*Behaviors*. Behaviors measured included [21]: (1) sleep duration via average number of hours slept within a 24 h period (continuous); (2) recent physical activity levels via total minutes of physical activity and exercise in the past week (continuous); (3) recent alcohol use via alcoholic beverage consumption in the past week (no, yes); (4) recent tobacco use via any tobacco product use (e.g., combustible cigarettes, smokeless tobacco) in the past 30 days (no, yes); and (5) recent cannabis use via cannabis in the past 30 days (no, yes).

#### 2.2.3. Patient Context—Healthcare Utilization

*Preventive Health Screenings, Testing, and Vaccinations*. Preventive health screenings in the past year included blood cholesterol test, blood pressure test, colon cancer test, blood sugar test, eye exam, dental exam, and routine check-up with physician [21]. Ever having prostate-specific antigen (PSA) testing and a sigmoidoscopy or colonoscopy were also measured [21]. Preventive vaccination history obtained included receiving a flu vaccine in the past year and a tetanus vaccine in the past 10 years [21].

*Hospitalization and Emergency Room Visits*. Participants were asked to report whether they had an overnight hospital stay or emergency room visit in the past 12 months [21].

*Falls*. Falls were measured by asking how many times participants have fallen in the past year [21], which was recategorized as 0, 1, and ≥2 falls.

#### 2.2.4. Informed, Activated Patient

*Preferred Method of Receiving Reliable Health/Medical Information*. One question asked whether participants preferred receiving reliable health/medical information about health or chronic conditions by either contacting a medical professional, finding it on the internet, or some other way [22,23].

*Frequency and Reliance of Help and Support Needed to Improve Health and Manage Health Problems*. The frequency of participants receiving the health and support needed to improve their health and manage health problems was measured using a 5-point scale ranging from never to always [22,23]. Due to the skewed nature of the responses, these were collapsed into never/rarely/occasionally versus frequently/always.

Reliance on receiving the ongoing help and support needed to improve their health and manage health problems was assessed using a 5-point scale ranging from: not at all to a great deal [22,23]. This scale was used to determine the level of reliance on: (1) their spouse or partner; (2) friends or relatives; (3) people with similar health problems; (4) co-workers; (5) doctors, nurses, or other healthcare providers; (6) church, synagogue, or other faith-based organizations; (7) community groups or clubs; and (8) the internet.

*Healthcare Frustrations Scale*. The healthcare frustrations scale is comprised of six questions that assessed whether participants felt any of the following frustrations by using the response options of never, occasionally, or frequently [22,23]: (1) felt tired of describing their same conditions and problems every time they go to a hospital or doctor’s office; (2) left the hospital or doctor’s office and felt confused about what they should do; (3) wished their doctor had more time to spend talking with them; (4) felt tired of feeling on their own when it comes to taking care of their health problems; (5) felt that their doctor does not realize what it is really like for them at home trying to take care of their health problems; and (6) wished they had a friend or family member who could go to the doctor with them. Scores for this scale ranged from 6 to 18, with higher scores translating to higher healthcare frustrations [30]. The Cronbach’s alpha scale value was acceptable for the current sample (α = 0.856).

*Disease Self-Management Efficacy Scale*. The disease self-management efficacy scale is comprised of the following 10 items that participants agreed or disagreed with using a 4-point scale [24,25], with response options of strongly disagree, disagree, agree, and strongly agree: (1) when all is said and done, they are responsible for taking care of their health; (2) taking an active role in their own health is the most important thing that affects their health; (3) they know what each of their prescription medication does; (4) they are confident that they can tell whether they need to go see the doctor or whether they can take care of a health problem themselves; (5) they are confident they can tell a doctor concerns they have even if he/she does not ask; (6) they are confident they can follow through on medical treatments they may need to do at home; (7) they have been able to maintain lifestyle changes, like eating right or exercising; (8) they know how to prevent problems with their health; (9) they are confident they can figure out solutions when new health problems arise with their health; and (10) they are confident that they can maintain lifestyle changes like eating right and exercising, even during times of stress. Scores for this scale ranged from 4 to 40, with higher scores translating to higher efficacy. The Cronbach’s alpha scale value was acceptable for the current sample (α = 0.844).

#### 2.2.5. Healthcare Barriers

*Barriers to Self-Care Scale*. The barriers to self-care scale is comprised of the following five items that participants agreed or disagreed with using a 4-point scale [22,23], with response options of strongly disagree, disagree, agree, and strongly agree: (1) they need help learning what they should be doing to take better care of their health; (2) they need help learning how to take better care of their health in a way that works for them and their life; (3) they do not have the money it takes to do things that will improve their health or condition; (4) they wish they could change and do things that are healthier, but they just do not think they can; and (5) all of their different health problems and conditions make it difficult for them to take better care of themselves. Scores for this scale ranged from 5 to 20, with higher scores translating to more barriers. The Cronbach’s alpha scale value was acceptable for the current sample (α = 0.844).

*Lack of Access to Medical Care and Medications Due to Costs*. Two questions assessed whether participants needed (1) a physician or (2) medications in the past year but could not receive the care or medications needed because of cost (no, yes) [21,22,23].

*Lack of Access to Medical Care Due to Other Reasons*. In addition to being asked about cost barriers to care, participants were asked about other reasons why they did not access medical care using the following “check all that apply” list: (1) could not get through on the phone; (2) could not get an appointment soon enough; (3) once there, had to wait too long to see the doctor; (4) the clinic or doctor’s office was not open when they arrived; and (5) did not have transportation [22,23].

#### 2.2.6. Productive Interactions

*Communication during Physician Visit Scale*. The communication during the physician visit scale is comprised of four questions related to when participants visit the doctor [24,25], how often they do the following using a 5-point scale with response options of never, almost never, sometimes, fairly often, and always: (1) prepared a list of questions for their doctor; (2) asked questions about things they want to know and things they do not understand about their treatment; (3) discussed any personal problems that may be related to their illness; and (4) asked questions until they clearly understand the purpose for taking each of their medications. Scores for this scale ranged from 4 to 20, with higher scores translating to higher engagement by the participants. The Cronbach’s alpha scale value was acceptable for the current sample (α = 0.810).

*Physician Quality Conversation and Joint Decision-Making Scale*. The physician quality conversation and joint decision-making scale is comprised of six questions about participants’ conversations with their healthcare providers [22,23], which asked how often their healthcare providers do the following using a 5-point scale with response options ranging from never to always: (1) asked for their ideas about how they can take care of their health problems; (2) made plans to contact them after a visit to see how they are doing; (3) helped them get the appointments they need; (4) asked if they understood their medications when prescribed such as how and when to take them, possible side effects, and drug interactions; (5) talked to other doctors and nurses who are taking care of them; and (6) asked whether they had help at home to manage their health problems. Scores for this scale ranged from 6 to 30, with higher scores translating to higher quality conversation [22,23]. The Cronbach’s alpha scale value was acceptable for the current sample (α = 0.846).

### 2.3. Statistical Analyses

For this descriptive study, all statistical analyses were performed using SPSS version 28. In addition to overall descriptives, each study variable was compared across a 4-item categorical variable created by combining race/ethnicity with age group. The four resulting categories were non-Hispanic Black men aged 40–64 (*n* = 933), non-Hispanic Black men aged ≥ 65 (*n* = 267), Hispanic men aged 40–64 (*n* = 625), and Hispanic men aged ≥ 65 (*n* = 203). A series of bivariate comparisons were made, with Cramer’s V tests used to identify associations between nominal variables. One-way ANOVAs were used to compare mean differences for continuous variables, and Tukey’s post hoc tests were performed to identify the categories with significant differences (signified with footnotes in the tables). Based on the exploratory nature of this study, and the large number of tests performed (*n* = 79), Bonferroni correction was applied. As such, the resulting *p*-value of 0.0006329 was used to determine statistical significance and account for potential type 2 errors.

## 3. Results

### 3.1. Patient Context—Sociodemographics

Of the 2028 participants, 59.2% self-identified as non-Hispanic Black and 40.8% self-identified as Hispanic. As seen in Table 1, the average age of participants was 56.54 (±10.03), with 23.2% being age ≥ 65 years. Approximately 20% of participants had a high school or less education and 37.3% had a 4-year college degree or more. Over half of the participants (52.2%) were married or partnered, 25% were never married, 18.9% were divorced or separated, and 3.8% were widowed. On average (±SD), participants reported 2.62 (±1.64) persons living in their home, including themselves. The majority of participants self-identified as straight or heterosexual (89.9%) and had health insurance coverage (89.0%). Over 29% of participants reported an annual household income of ≤$29,999, with 39.7% reporting ≥$60,000. Approximately 30% of participants reported past or current service in the U.S. armed forces and resided in a metro area (93.7%).

Stratified by racial/ethnic age groups, there were significant differences based on all sociodemographic variables. Concerning age, the mean age was lower among the Hispanic younger age group followed by the non-Hispanic Black younger age group, while the mean age was higher among the Hispanic older age group than the non-Hispanic Black older age group (*p* < 0.0001). Education level (*p* < 0.0001) and marital status (*p* < 0.0001) both significantly varied. The non-Hispanic Black younger age group reported the highest percentages of obtaining ≤high school education (23.6%) or some college/2-year degree (46.3%) and never being married (35.8%), while the Hispanic older age group reported the highest percentages of obtaining a ≥4-year degree (60.1%) and being married/partnered (73.4%). The non-Hispanic Black older age group had the highest percentages of being divorced/separated (23.2%) or being widowed (10.9%). While the majority of men across racial/ethnic and age groups were straight/heterosexual, the Hispanic younger group had significantly higher reports of identifying as gay/homosexual (7.8%), bisexual (5.0%), or identifying in some other way (1.3%; *p* = 0.001). Both the Hispanic and non-Hispanic Black older age groups had the highest reports of living in a metro area (96.1% and 95.5%), having health insurance coverage (97.0% and 95.9%), and past/current service in the U.S. armed services (38.4% and 46.8%), respectively. The Hispanic and non-Hispanic Black younger age groups had a significantly higher mean number of persons living in their household including themselves (*p* < 0.0001) and the highest report of an annual household income of <$10,000 (7.0% and 7.2%, *p* < 0.0001) (see Table 1).

### 3.2. Patient Context—Health Status

As seen in Table 2, on average, participants self-reported 4.01 (±2.98) chronic health conditions and taking 3.39 (±2.02) prescription medications daily. The most prevalent reported chronic health conditions were hypertension (55.9%), high cholesterol (45.4%), diabetes (37.9%), chronic pain (36.8%), depression or anxiety (31.9%), and arthritis/rheumatic disease (30.3%).

Stratified by racial/ethnic age groups, significant differences were found based on 3 of the 19 chronic health conditions assessed including cancer or cancer survivor (*p* < 0.0001), depression or anxiety (*p* < 0.0001), and hypertension (*p* < 0.0001). The non-Hispanic Black younger age group had the highest prevalence of osteoporosis (8.4%) and schizophrenia or other psychotic disorder (8.1%), while the Hispanic younger age group had the highest prevalence of depression or anxiety (38.4%). The non-Hispanic Black and Hispanic younger age groups had similar high, respective reports of chronic pain (40.0% and 38.1%), obesity (24.5% and 26.4%), and asthma/emphysema/chronic breathing or a lung problem (20.5% and 20.3%). 

The non-Hispanic Black older age group had the highest prevalence of having hypertension (73.8%) and cancer or being a cancer survivor (29.2%), while the Hispanic older age group had the highest prevalence of diabetes (46.8%), heart disease (21.2%), and a thyroid problem (12.8%). The non-Hispanic Black and Hispanic older age groups had similar reports of high cholesterol (53.9% and 52.7%, respectively). While having lower prevalence than the younger age groups, the non-Hispanic Black and Hispanic older age groups had similar high reports of having chronic pain (29.6% and 28.1%) and depression and anxiety (18.0% and 21.7%). Obstructive sleep apnea was highest among the Hispanic younger (25.8%) and older (25.6%) age groups. Daily medications taken was highest among both older age groups (*p* < 0.0001; see Table 2).

The participants’ overall mean of general health status was 2.84 (±0.89). The four racial/ethnic age groups had similar reported mean general health status, but the poorest health status was reported among the non-Hispanic Black younger age group. The overall mean of participants’ fatigue and quality of life scores were 3.59 (±3.29) and 6.90 (±1.94), respectively. The non-Hispanic Black and Hispanic younger age groups had similar lower average scores on the fatigue and quality of life health indicator scales. The overall mean of participants’ stress, sleep problems, and pain health scores were 3.69 (±3.35), 3.95 (±3.36), and 4.10 (±3.33), respectively. The non-Hispanic Black and Hispanic older age groups had similar lower averages on stress, sleep problems, and pain health indicator scales (all *p* < 0.0001). 

Approximately 32% of participants scored past the threshold for depressive symptomatology. The prevalence of depressive symptomatology was significantly higher among the Hispanic younger age group (38.4%), followed by the non-Hispanic Black younger age group (33.8%), Hispanic older age group (21.7%), and non-Hispanic Black older age group (18.0%; *p* < 0.0001).

Participants reported an average of 6.62 (±1.73) number of hours slept within a 24 h period and an average of 147.21 (±170.27) total minutes of physical activity in the past week (see Table 3). Concerning substance use, 61.4% recently used alcohol, 35.2% recently used tobacco, and 21.7% recently used cannabis. Group differences were found on these behaviors. Specifically, the non-Hispanic Black and Hispanic younger age groups reported lower mean sleep duration (*p* < 0.0001), but higher prevalence of recent tobacco use (42.3% and 34.6%, *p* < 0.001) and recent cannabis use (26.8% and 21.9%; *p* < 0.0001), respectively. The non-Hispanic Black younger age group had the lowest average minutes of physical activity and highest prevalence of recent alcohol use (64.0%), and both Hispanic age groups had similar reports of average total minutes of physical activity and recent alcohol use.

### 3.3. Patient Context—Healthcare Utilization

As seen in Table 3, between 31.0% and 87.4% of participants reported engaging in any given preventive health screening in the past year. With the exception of flu vaccinations (*p* = 0.0007) and colon cancer testing (*p* < 0.0001), both older age groups had a significantly high prevalence of having preventive health screenings, testing, and vaccinations compared with the younger age groups. Specifically, the non-Hispanic Black and Hispanic older age groups had similar respective reports of having: a blood pressure test (96.3% and 97.0%), routine check-up with a physician (92.9% and 91.6%), blood cholesterol test (83.1% and 90.6%), blood sugar test (77.5% and 84.2%), sigmoidoscopy or colonoscopy (81.3% and 81.3%), PSA test (80.9% and 79.8%), eye exam (66.3% and 73.9%), dental exam (59.9% and 66.5%), receiving a tetanus shot (64.0% and 63.1%), and receiving a flu vaccine (49.4% and 48.8%). Less than 20% of participants attended a program to prevent or manage their chronic condition(s) in the past year, with younger groups across race/ethnicity attending at a higher rate.

A total of 27.8% and 44.4% of participants reported ≥1 overnight hospital stay and emergency room visit in the past year, respectively (see Table 2). The prevalence of having an overnight hospital stay and emergency room visit were highest among the non-Hispanic Black (30.2% and 48.6%) and Hispanic (29.4% and 45.9%) younger age groups followed by the non-Hispanic Black (22.8% and 38.6%) and Hispanic (17.7% and 28.6%) older age groups, respectively.

Overall, 11.4% of participants reported one fall and 19.8% reported ≥2 falls. The highest prevalence of having ≥2 falls was among the Hispanic (22.6%) and non-Hispanic Black (21.2%) younger age groups followed by the Hispanic (17.2%) and non-Hispanic Black (10.1%) older age groups.

### 3.4. Informed, Activated Patient

As seen in Table 4, most participants preferred receiving reliable health/medical information about their health or chronic conditions by contacting a medical professional (70.7%), followed by the internet (27.6%) and some other way (1.8%). The non-Hispanic Black and Hispanic older age groups (80.1% and 75.4%) reported high preference to contact a medical professional, whereas the non-Hispanic Black and Hispanic younger groups reported high preference of using the internet (26.8% and 33.9%), respectively.

Approximately 43% of participants reported they never/rarely/occasionally received the health and support needed to improve their health and manage health problems, with the highest reliance on their doctors, nurses, or other healthcare providers, spouse or partner, and internet. The non-Hispanic Black and Hispanic younger age groups had similar average report of relying on the internet, friends or relatives, people with similar health problems, church synagogue or other faith-based organizations, community groups or clubs, and co-workers. Although the highest report of relying on a spouse or partner was among both Hispanic age groups, both non-Hispanic Black age groups had comparable average reports of relying on their spouse or partner. The non-Hispanic Black younger age group and both Hispanic age groups had similar average reports of relying on friends or relatives. All four groups reported a high average of relying on doctors, nurses, or other healthcare providers.

The overall mean scores of healthcare frustrations and disease self-management efficacy were 9.53 (±3.15) and 28.48 (±3.65), respectively. The non-Hispanic Black and Hispanic younger groups had higher levels of healthcare frustrations (*p* < 0.0001) while the two older groups had higher disease self-management efficacy.

### 3.5. Healthcare Barriers

The overall mean score of barriers to self-care was 11.53 (±3.65) and the non-Hispanic Black and Hispanic younger groups had higher barriers to self-care (*p* < 0.0001). Nearly one-fifth (19.1%) of participants needed a physician and 21.3% needed medications in the past year but could not receive the care or medications needed because of cost. The non-Hispanic Black and Hispanic younger age groups had the highest prevalence of not receiving the physician care (21.4% and 25.9%, *p* < 0.0001) or medications needed (25.2% and 24.7%, *p* < 0.0001) due to cost, respectively.

The most common reason why participants did not access medical care was that nearly half (49.7%) could not get an appointment soon enough, followed by 21.6% having to wait too long to see the doctor, 19.4% did not have transportation, 19.1% could not get through on the telephone, and 7.3% reported the office was not open when they got there. Not having transportation was the only significant difference found based on other reasons, with a higher prevalence among the non-Hispanic Black younger age group (21.7%), followed by the Hispanic younger age group (20.6%), non-Hispanic Black older age group (19.9%), and Hispanic older age group (4.9%; see Table 4).

### 3.6. Productive Interactions

The overall mean scores of communication during the physician visit and physician quality conversation and joint decision making were 14.10 (±3.55) and 18.64 (±5.55). The mean scores of communication during the physician visit and physician quality conversation and joint decision making were relatively higher among both non-Hispanic Black age groups (see Table 4).

## 4. Discussion

Framed by a modified CCM accounting for patient context, the present study utilized self-reported data to describe the health and experiences of non-Hispanic Black and Hispanic men living with chronic conditions related to disease management. Several results by age group merit further attention. While participants’ sociodemographics differed by race/ethnicity more so than by age, the younger age groups, irrespective of race/ethnicity, reported a lower prevalence of living in a metro area, a lower prevalence of having health insurance coverage, had lower income, and lived with more people on average. Such physical and social contextual information about this younger age group provides initial insight into the additional structural influences that may influence their ability to take care of their own health [31,32].

With the exception of obstructive sleep apnea, which was highest among both Hispanic male groups, in general, chronic health conditions differed by age. Racial/ethnic older age groups reported greater physical health conditions such as a higher prevalence of hypertension, high cholesterol, and diabetes, and took more prescribed medications than younger age groups. In contrast, younger age groups reported greater mental health conditions including depression, anxiety, schizophrenia, and chronic pain. Some of these age differences may be explained by older men’s greater access and use of preventive health screenings, testing and vaccinations that are offered free through Medicare in the U.S. [33]. For example, after being screened for diabetes or high cholesterol, older men can use Medicare Part D to cover their prescription drugs to treat their health condition. Older men may also be screened for mental health issues, such as depression, and receive appropriate treatment.

Conversely, younger age groups in our study reported a lower prevalence of having routine check-ups and eye and dental exams in the past year, which represent opportunities for healthcare professionals to detect underlying health conditions such as chronic pain and associated mental health disorders. The lack of access to preventive care among younger age groups was compensated by seeking care when experiencing more severe conditions, as explained by the higher prevalence of two or more falls in the past year and the greater incidence of emergency room visits and hospitalizations compared to older age groups. Using acute hospital care and less primary care is common among vulnerable population groups, such as patients with low socioeconomic status [34].

Economic access to healthcare also disproportionately affected the younger age groups in our study. The costs associated with visiting a physician and receiving prescribed medications as well as having transportation to get access to the care they needed (e.g., primarily for those living in rural areas) were identified as barriers to preventive care access. This finding is also supported by previous studies on the experience of racially/ethnically diverse populations seeking healthcare [35,36,37].

As indicated previously, the younger age groups also had a greater prevalence of being uninsured. A study by Sohn [38] reported a high prevalence of lack of insurance or unstable health insurance coverage among non-Hispanic Black and Hispanic populations between the ages of 40 and 60 years because of their employment opportunities and their eligibility for and/or the affordability of health benefits. Unstable insurance coverage can affect the healthcare younger age groups receive.

Interestingly, our study findings suggest that non-Hispanic Black and Hispanic younger men may compensate for their lack of access to preventive care by preferring to receive health information on the internet [39]. Madrigal and Escoffery [40] indicated that participants with chronic diseases found online health information to be useful and important to learn about, manage, and cope with a chronic disease. Gordon and Crouch [41] found that middle-aged adults with chronic conditions preferred accessing and receiving internet-based health information more often than older age groups. More research is needed on the use of online health information specifically by non-Hispanic Black and Hispanic men with chronic conditions.

Compared to both the non-Hispanic Black and Hispanic older age groups, younger age groups had higher levels of healthcare frustrations and barriers to self-care, while having lower disease self-management efficacy. Racially/ethnically diverse populations, including non-Hispanic Black and Hispanic men, have previously reported experiencing greater healthcare-related frustrations and barriers to self-care compared to non-Hispanic Whites [30,42]. However, when enrolled in a chronic disease self-management education program, non-Hispanic Black and Hispanic men were more likely than non-Hispanic White men to complete the intervention and develop self-efficacy [43]. Older men aged 65 to 79 were also significantly more likely to complete the program than younger age groups, as they may be more open to gaining the knowledge and skills to manage their chronic condition [43]. In our study, close to one-in-four non-Hispanic Black and Hispanic younger men were also informal caregivers, which may contribute to their increased stress and depressive symptoms [44] and the neglect of their own health needs [44,45].

Compared to older age groups, non-Hispanic Black and Hispanic men from younger age groups engaged in less healthy behaviors. On average, they did not meet sleep recommendations for their age group of seven or more hours per night [46], had the highest cannabis use, and significantly exceeded the national average for tobacco use [47]. Engaging in risky health behaviors such as smoking and drug use are common mechanisms used to cope with life stressors such as low socioeconomic status, lack of health insurance, and caregiver duties [48]. These health behaviors may exacerbate other conditions such as depression and anxiety, which can lead to more risky behaviors [49] and increase the risks of comorbidities in older adulthood [50].

These findings in the context of the CCM may guide opportunities for interventions in healthcare and community settings. As mentioned, the non-Hispanic Black and Hispanic younger men groups had disproportionately worse health status (e.g., stress, sleep problems, pain, depressive symptomatology, risky lifestyle behaviors), more hospitalizations, more emergency room visits, and less preventive screening. Further, non-Hispanic Black and Hispanic younger men were less informed, activated patients in that they received less help/support to improve or manage their conditions, had more healthcare-related frustrations, and worse disease self-management efficacy. Additionally, the younger men reported more barriers to self-care. Despite more disease symptomatology, risk, and barriers, no more than 20.2% of these younger men attended a program to prevent or manage their chronic condition(s). As such, effective prevention and management programs to enhance self-management and promote healthy behaviors among non-Hispanic Black and Hispanic younger men with chronic conditions are key to reducing racial/ethnic health disparities. For example, interventions such as the Workplace Chronic Disease Self-Management Program [51,52] can be offered to working-aged men to improve access to community resources for self-management support, promote more productive interactions with healthcare providers, and ultimately increase patient activation while decreasing disease symptomatology (see CCM components in Figure 1). The Centers for Disease Control and Prevention’s Racial and Ethnic Approaches to Community Health (REACH) Program [53] provides funding to support culturally-tailored interventions across the U.S. to reduce tobacco use, poor nutrition, and physical inactivity, while also increasing community-clinical linkages for enhanced disease management. Interventions specific to non-Hispanic Black and Hispanic men, such as Animo, the weight loss pilot randomized controlled trial for Hispanic men [54], are strongly encouraged. Further, because more younger men in this study preferred getting their health information online and received support from more sources, the use of technology (e.g., internet, telehealth, health apps, and text messages) and different settings (e.g., faith-based organizations, workplaces, senior centers) for health information sharing and interventions are also recommended [55,56,57,58].

This study is not without limitations. While the value of this paper is its focus on non-Hispanic Black and Hispanic men with chronic conditions, it does not provide an overview of health indicators and healthcare utilization for men from other racial/ethnic groups (e.g., Asian, American Indian and Alaska Natives, Native Hawaiian or Pacific Islander) or of racially/ethnically diverse women. A similar study is recommended to capture the realities and factors influencing the health of these other racial and ethnic groups. Considering that chronic conditions increase with age [2], the current (≥65 years) and next generation of older adults (40–64 years) were selected and compared for this study; however, a longer life course perspective would have provided greater insight on upstream preventative efforts needed to reduce health disparities among non-Hispanic Black and Hispanic men. Data were self-reported and cross-sectional in nature, which may introduce reporting bias and limit causal inferences. While many elements of the CCM were included in this study, measures related to the health system in which these men interact (including the preparedness and proactive nature of the practice team) were not assessed. Data were collected using an internet-delivered questionnaire, which may have introduced some bias in that not all possible participants had access to the internet. Further, it is possible that the sample obtained online may be of higher socioeconomic statuses based on their employment or internet access. For the purposes of this study, statistical analyses were limited to bivariate comparisons. The number of comparisons may have introduced some bias and measurement error. However, this study serves as an important first step to drive subsequent analyses and predictive modeling using this sample. Additional analyses are recommended to further examine relationships between each racial/ethnic age group and the different health indicators.

## 5. Conclusions

Overall, this study provides a strong overview of the patient context as it relates to perceptions about being an informed, activated patient and having productive interactions in healthcare settings among non-Hispanic Black and Hispanic men with chronic conditions. This study provides a unique glimpse into the factors contributing to health and wellness among these understudied subgroups in the U.S. Racial/ethnic age differences may help inform future prevention and management interventions specific to non-Hispanic Black and Hispanic men.

## Figures and Tables

**Table 1:** Patient context: Sociodemographics by race/ethnicity and age.

	Total	Non-Hispanic Black		Hispanic			
	(*n* = 2028)	Age 40–64 (*n* = 933)	Age ≥ 65 (*n* = 267)	Age 40–64 (*n* = 625)	Age ≥ 65 (*n* = 203)	V or f	*p*
***Age***	56.54 (±10.03)	52.98 (±7.23)	69.90 (±4.36)	51.60 (±7.24)	70.26 (±4.71)	837.80 ^a^	<0.0001
***Education***						0.131	<0.0001
≤High School Graduate	20.3%	23.6%	18.0%	19.5%	10.8%		
Some College or 2-Year Degree	42.4%	46.3%	42.7%	40.6%	29.1%		
≥4-Year Degree	37.3%	30.1%	39.3%	39.8%	60.1%		
***Marital Status***						0.176	<0.0001
Married or Partnered	52.2%	42.1%	55.1%	59.0%	73.4%		
Never Married	25.0%	35.8%	10.9%	21.6%	4.9%		
Divorced or Separated	18.9%	19.5%	23.2%	17.0%	16.7%		
Widowed	3.8%	2.6%	10.9%	2.4%	4.9%		
***Persons Living in Household (including Self)***	2.62 (±1.64)	2.64 (±1.76)	2.06 (±1.29)	2.95 (±1.67)	2.17 (±0.94)	24.57 ^b^	<0.0001
***Sexual Orientation***						0.069	0.001
Straight or Heterosexual	89.9%	90.7%	96.6%	85.9%	90.1%		
Gay or Homosexual	6.2%	6.3%	2.2%	7.8%	5.9%		
Bisexual	3.2%	2.5%	1.1%	5.0%	3.4%		
Identify in Some Other Way	0.7%	0.5%	0.0%	1.3%	0.5%		
***Annual Household Income***						0.123	<0.0001
Less than $10,000	5.9%	7.2%	2.2%	7.0%	1.0%		
$10,000 to $19,999	10.6%	12.3%	11.6%	7.7%	9.9%		
$20,000 to $29,999	12.9%	14.0%	12.7%	12.0%	10.3%		
$30,000 to $39,999	10.9%	12.1%	14.6%	9.1%	5.9%		
$40,000 to $49,999	9.3%	9.4%	9.7%	9.3%	8.4%		
$50,000 to $59,999	10.7%	11.5%	7.5%	10.9%	11.3%		
$60,000 to $69,999	6.3%	5.8%	7.9%	6.4%	6.4%		
$70,000 to $79,999	7.5%	7.5%	8.6%	6.4%	9.4%		
$80,000 to $89,999	3.6%	3.1%	2.6%	4.5%	4.4%		
$90,000 to $99,999	3.8%	2.1%	3.4%	5.9%	5.9%		
$100,000 to $149,999	11.5%	9.9%	12.7%	13.0%	12.8%		
$150,000 or More	7.0%	5.0%	6.4%	7.8%	14.3%		
***Insurance Coverage***						0.132	<0.0001
No/Don’t Know	11.0%	12.6%	4.1%	14.2%	3.0%		
Yes	89.0%	87.4%	95.9%	85.8%	97.0%		
***Past/Current Service in U.S. Armed Services***						0.185	<0.0001
No	70.4%	71.3%	53.2%	79.2%	61.6%		
Yes	29.6%	28.7%	46.8%	20.8%	38.4%		
***Rurality***						0.066	0.033
Metro	93.7%	92.1%	95.5%	94.7%	96.1%		
Non-Metro	6.3%	7.9%	4.5%	5.3%	3.9%		

Superscripts represent significant differences for continuous variables based on Tukey’s post hoc tests, which are only presented for statistically significant differences based on applied Bonferroni corrections. ^a^ = difference between age categories; ^b^ = difference between age and race/ethnicity categories.

**Table 2:** Patient context: Health status by race/ethnicity and age group.

	Total	Non-Hispanic Black		Hispanic			
	(*n* = 2028)	Age 40–64 (*n* = 933)	Age ≥ 65 (*n* = 267)	Age 40–64 (*n* = 625)	Age ≥ 65 (*n* = 203)	V or f	*p*
***Chronic Conditions***	4.01 (±2.98)	4.04 (±3.06)	3.93 (±2.50)	3.96 (±3.10)	4.09 (±2.81)	0.210	0.890
Asthma/Emphysema/Chronic Breathing or Lung Problem	18.8%	20.5%	15.0%	20.3%	11.8%	0.076	0.009
Arthritis/Rheumatic Disease	30.0%	32.2%	28.1%	26.6%	33.0%	0.058	0.074
Cancer or Cancer Survivor	14.4%	13.1%	29.2%	9.3%	17.2%	0.177	<0.0001
Chronic Pain	36.8%	40.0%	29.6%	38.1%	28.1%	0.092	0.001
Depression or Anxiety	31.9%	33.8%	18.0%	38.4%	21.7%	0.153	<0.0001
Diabetes	37.9%	35.3%	42.3%	37.1%	46.8%	0.077	0.008
Heart Disease	13.0%	11.1%	12.0%	13.4%	21.2%	0.087	0.002
High Cholesterol	45.4%	42.2%	53.9%	44.2%	52.7%	0.090	0.001
Hypertension (High Blood Pressure)	55.9%	56.6%	73.8%	45.0%	62.6%	0.184	<0.0001
Kidney Disease	8.1%	9.0%	7.1%	7.0%	8.4%	0.034	0.506
Memory Problem (e.g., dementia, Alzheimer’s disease)	5.8%	5.7%	4.1%	6.7%	5.9%	0.034	0.500
Obesity	23.5%	24.5%	15.0%	26.4%	21.2%	0.086	0.002
Osteoporosis (Low Bone Density)	6.6%	8.4%	2.6%	6.4%	4.4%	0.080	0.004
Obstructive Sleep Apnea (snoring or trouble breathing when sleeping)	22.9%	22.6%	15.0%	25.8%	25.6%	0.081	0.004
Schizophrenia or Other Psychotic Disorder	6.6%	8.1%	4.1%	6.4%	3.4%	0.069	0.022
Stroke	7.1%	8.0%	6.0%	6.9%	4.9%	0.040	0.356
Thyroid Problem (e.g., Hyperthyroidism, Hypothyroidism)	8.9%	6.9%	8.2%	10.9%	12.8%	0.076	0.008
Urinary Incontinence	9.8%	9.2%	13.9%	8.3%	11.8%	0.062	0.050
Other Chronic Condition	17.1%	17.0%	15.0%	18.7%	15.3%	0.035	0.478
***Number of Medications Taken Daily (0 to ≥6)***	3.39 (±2.02)	3.23 (±2.05)	4.22 (±1.76)	3.04 (±1.97)	4.14 (±1.85)	34.19 ^b^	<0.0001
***General Health Status (1* = *poor; 5* = *excellent)***	2.84 (±0.89)	2.80 (±0.90)	2.88 (±0.79)	2.82 (0.90±)	3.02 (±0.89)	3.75	0.011
***Disease Symptoms***							
Fatigue (0 to 10)	3.59 (±3.29)	3.71 (±3.30)	2.51 (±3.02)	4.21 (±3.32)	2.51 (±2.85)	25.70 ^a^	<0.0001
Pain (0 to 10)	4.10 (±3.33)	4.24 (±3.35)	3.18 (±3.23)	4.62 (±3.33)	3.00 (±2.89)	20.55 ^b^	<0.0001
Shortness of Breath (0 to 10)	2.30 (±2.99)	2.35 (±3.05)	2.04 (±2.87)	2.63 (±3.10)	1.37 (±2.26)	9.93 ^b^	<0.0001
Stress (0 to 10)	3.69 (±3.35)	4.06 (±3.41)	1.96 (±2.60)	4.40 (±3.30)	2.08 (±2.77)	56.69 ^a^	<0.0001
Sleep Problem (0 to 10)	3.95 (±3.36)	4.13 (±3.34)	2.52 (±3.01)	4.69 (±3.34)	2.73 (±3.01)	38.13 ^a^	<0.0001
***Depressive Symptomatology***						0.212	<0.0001
No	68.1%	66.2%	82.0%	61.6%	78.3%		
Yes	31.9%	33.8%	18.0%	38.4%	21.7%		
***Behavior***							
Average Hours of Sleep in 24 Hour Period	6.62 (±1.73)	6.49 (±1.85)	6.97 (±1.86)	6.48 (±1.57)	7.18 (±1.27)	14.25 ^a^	<0.0001
Total Minutes of Physical Activity (past week)	147.21 (±170.27)	133.34 (±164.60)	145.32 (±152.91)	160.16 (±172.91)	171.21 (±197.91)	3.28	0.020
Weekly Alcoholic Beverage Consumption	61.4%	64.0%	54.7%	60.5%	61.6%	0.063	0.047
Tobacco Use in Past 30 Days	35.2%	42.3%	29.2%	34.6%	11.8%	0.191	<0.0001
Cannabis Use in Past 30 Days	21.7%	26.8%	13.1%	21.9%	9.4%	0.147	<0.0001

Superscripts represent significant differences for continuous variables based on Tukey’s post hoc tests, which are only presented for statistically significant differences based on applied Bonferroni corrections. ^a^ = difference between age categories; ^b^ = difference between age and race/ethnicity categories.

**Table 3:** Patient context: Healthcare utilization by race/ethnicity and age group.

	Total	Non-Hispanic Black		Hispanic			
	(*n* = 2028)	Age 40–64 (*n* = 933)	Age ≥ 65 (*n* = 267)	Age 40–64 (*n* = 625)	Age ≥ 65 (*n* = 203)	V or f	*p*
***Preventive Screening***							
Flu Vaccine in Past Year	41.4%	41.3%	49.4%	35.8%	48.8%	0.098	0.0002
Tetanus Shot in Past 10 Years	58.0%	54.7%	64.0%	58.9%	63.1%	0.072	0.014
Blood Cholesterol Test Past Year	75.8%	74.4%	83.1%	70.1%	90.6%	0.148	<0.0001
Blood Pressure Test Past Year	87.4%	86.3%	96.3%	82.1%	97.0%	0.162	<0.0001
Colon Cancer Test Past Year	31.0%	32.7%	39.0%	24.6%	32.5%	0.102	<0.0001
Blood Sugar Test Past Year	71.4%	70.2%	77.5%	66.6%	84.2%	0.120	<0.0001
Eye Exam Past Year	58.0%	54.0%	66.3%	55.4%	73.9%	0.134	<0.0001
Dental Exam Test Past Year	54.4%	50.2%	59.9%	54.6%	66.5%	0.104	<0.0001
Prostate-Specific Antigen (PSA) Test in Lifetime	54.7%	49.7%	80.9%	42.9%	79.8%	0.290	<0.0001
Sigmoidoscopy or Colonoscopy in Lifetime	56.7%	52.7%	81.3%	44.0%	81.3%	0.283	<0.0001
Routine Check-Up with Physician in Past Year	84.6%	84.9%	92.9%	78.4%	91.6%	0.141	<0.0001
Ever Attend Program to Prevent or Manage Chronic Illness in Past Year	17.9%	20.2%	15.4%	17.4%	12.8%	0.063	0.045
***Overnight Hospital Stay in Past Year***						0.092	0.0007
No	72.2%	69.8%	77.2%	70.6%	82.3%		
Yes	27.8%	30.2%	22.8%	29.4%	17.7%		
***Emergency Room Visit in Past Year***						0.124	<0.0001
No	55.6%	51.4%	61.4%	54.1%	71.4%		
Yes	44.4%	48.6%	38.6%	45.9%	28.6%		
***Falls in the Past Year***						0.089	<0.0001
None	68.8%	69.3%	77.9%	65.3%	65.0%		
Once	11.4%	9.4%	12.0%	12.2%	17.7%		
Twice or More	19.8%	21.2%	10.1%	22.6%	17.2%		

**Table 4:** Chronic Care Model: Patient activation, healthcare barriers, and productive interactions by race/ethnicity and age group.

	Total	Non-Hispanic Black		Hispanic			
	(*n* = 2028)	Age 40–64 (*n* = 933)	Age ≥ 65 (*n* = 267)	Age 40–64 (*n* = 625)	Age ≥ 65 (*n* = 203)	V or f	*p*
**INFORMED, ACTIVATED PATIENT**							
***Preferred Method of Getting Reliable Health/Medical Information***						0.082	0.0001
Medical Professional	70.7%	71.1%	80.1%	64.5%	75.4%		
The Internet	27.6%	26.8%	18.7%	33.9%	23.2%		
Some Other Way	1.8%	2.1%	1.1%	1.6%	1.5%		
***Get the Help/Support Needed to Improve Health and Manage Health Problems***						0.202	<0.0001
Never/Rarely/Occasionally	42.9%	44.7%	25.5%	53.1%	26.6%		
Frequently/Always	57.1%	55.3%	74.5%	46.9%	73.4%		
***Reliance for Ongoing Help/Support to Improve Health and Manage Health Problems***							
Co-Workers	1.47 (±0.93)	1.50 (±0.97)	1.18 (±0.64)	1.62 (±1.01)	1.21 (±0.56)	20.65 ^b^	<0.0001
Community Groups or Clubs	1.52 (±0.99)	1.57 (±1.05)	1.37 (±0.79)	1.62 (±1.05)	1.17 (±0.50)	13.50 ^a^	<0.0001
Church, Synagogue, or Other Faith-Based Organizations	1.77 (±1.15)	1.85 (±1.22)	1.67 (±1.10)	1.80 (±1.16)	1.41 (±0.79)	8.82 ^a^	<0.0001
People with Similar Health Problems	1.78 (±1.06)	1.83 (±1.11)	1.60 (±0.90)	1.85 (±1.11)	1.57 (±0.80)	6.95 ^a^	<0.0001
Friends or Relatives	2.06 (±1.12)	2.08 (±1.13)	1.85 (±0.99)	2.15 (±1.16)	2.03 (±1.06)	4.68	0.003
Internet	2.28 (±1.20)	2.33 (±1.22)	1.90 (±0.98)	2.46 (±1.25)	2.00 (±1.07)	18.10 ^b^	<0.0001
Spouse or Partner	2.61 (±1.50)	2.41 (±1.46)	2.43 (±1.46)	2.84 (±1.54)	3.05 (±1.47)	17.98 ^b^	<0.0001
Doctors, Nurses, or Other Healthcare Providers	3.44 (±1.24)	3.48 (±1.25)	3.61 (±1.25)	3.25 (±1.24)	3.65 (±1.09)	9.00 ^a^	<0.0001
***Healthcare Frustrations*** (6 to 18, higher = more frustration)	9.53 (±3.15)	9.71 (±3.14)	8.23 (±2.61)	10.23 (±3.31)	8.24 (±2.30)	40.28^a^	<0.0001
***Disease Self-Management Efficacy*** (10 to 40, higher = more efficacy)	28.48 (±2.67)	28.35 (±2.95)	28.92 (±1.60)	28.44 (±2.35)	28.64 (±3.27)	3.40	0.017
**HEALTHCARE BARRIERS**							
***Barriers to Self-Care*** (5 to 20, higher = more barriers)	11.53 (±3.65)	11.77 (±3.62)	10.23 (±3.42)	12.25 (±3.62)	9.92 (±3.22)	35.77 ^a^	<0.0001
***Needed Physician in Past Year but Didn’t Go Because of Cost***						0.199	<0.0001
No	80.9%	78.6%	93.6%	74.1%	96.1%		
Yes	19.1%	21.4%	6.4%	25.9%	3.9%		
***Needed Medications in Past Year but Didn’t Because of Cost***						0.161	<0.0001
No	78.7%	74.8%	88.0%	75.3%	92.8%		
Yes	21.3%	25.2%	12.0%	24.7%	7.2%		
***Other Than Cost, Delayed Getting Medical Care Because***							
Couldn’t get through on the telephone	19.1%	18.5%	16.1%	19.8%	23.2%	0.045	0.249
Couldn’t get an appointment soon enough	49.7%	49.0%	43.8%	52.6%	51.2%	0.055	0.101
Once there, had to wait too long to see the doctor	21.6%	20.3%	20.6%	23.2%	24.6%	0.040	0.363
The clinic or doctor’s office wasn’t open when you got there	7.3%	7.3%	6.7%	7.5%	7.4%	0.009	0.982
Didn’t have transportation	19.4%	21.7%	19.9%	20.6%	4.9%	0.123	<0.0001
**PRODUCTIVE INTERACTIONS**							
***Communication During Physician Visit*** (4 to 20, higher = more engagement)	14.10 (±3.55)	14.33 (±3.49)	14.04 (±3.46)	13.82 (±3.60)	13.97 (±3.78)	2.72	0.043
***Physician Quality Conversation and Joint Decision Making*** (6 to 30, higher = more quality conversation)	18.64 (±5.55)	18.97 (±5.45)	19.28 (±5.72)	17.86 (±5.58)	18.67 (±5.43)	6.45 ^b^	<0.0001

Superscripts represent significant differences for continuous variables based on Tukey’s post hoc tests, which were only presented for statistically significant differences based on applied Bonferroni corrections. ^a^ = difference between age categories; ^b^ = difference between age and race/ethnicity categories.

## Data Availability

Restrictions apply to the availability of these data. Data was obtained through the State of Texas and may be available from the authors with the permission of the State of Texas.
